# Supplementary material for: How does serif vs sans serif typeface impact the usability of e-commerce websites?
Source: PeerJ Comput Sci. 2022 Nov 18;8:e1139. doi: 10.7717/peerj-cs.1139 (PMC9680897; doi:10.7717/peerj-cs.1139)
Supplement: Supplemental Information 2 [file peerj-cs-08-1139-s002.pdf]

Questionnaire

This is an anonymous questionnaire that gathers data for reading comprehension and the usability of the website. It only takes around 1 or 2 minutes. Thank you for your participation!

* Compulsory fields

Birthdate *

Day: Choose a day **Month:** Choose a month **Year:** Choose a year

Gender *

Choose your gender

How much time are potatoes boiled in order to become soft? *

- ○ Between 20 and 30 minutes
- ○ Between 10 and 25 minutes
- ○ Between 20 and 25 minutes

The website is easy to use *

Strongly disagree	○	○	○	○	○	Strongly agree
	1	2	3	4	5	

It is easy to navigate within the website *

Strongly disagree	○	○	○	○	○	Strongly agree
	1	2	3	4	5	

I feel comfortable purchasing from the website *

Strongly disagree	○	○	○	○	○	Strongly agree
	1	2	3	4	5	

I feel confident conducting business on the website *

Strongly disagree	○	○	○	○	○	Strongly agree
	1	2	3	4	5	

How likely are you to recommend this website to a friend or colleague? *

Not at all likely	○	○	○	○	○	○	○	○	○	○	○	Very likely
	0	1	2	3	4	5	6	7	8	9	10	

I will likely return to the website in the future *

Strongly disagree	○	○	○	○	○	Strongly agree
	1	2	3	4	5	

I find the website to be attractive *

Strongly disagree	○	○	○	○	○	Strongly agree
	1	2	3	4	5	

The website has a clean and simple presentation *

Strongly disagree	○	○	○	○	○	Strongly agree
	1	2	3	4	5	

It is easy to read the text on this website with the used font type *

Strongly disagree	○	○	○	○	○	Strongly agree
	1	2	3	4	5	
